# Identification and validation of prognostic characteristics of survival risk marker genes associated with ferroptosis and iron metabolism in breast cancer

**DOI:** 10.1590/1414-431X2026e15313

**Published:** 2026-10-30

**Authors:** Mengxi Li, Baoqing Zhao, Zhenwang Zhang

**Affiliations:** 1 Department of Biological Sciences, School of Nuclear Technology and Chemistry & Biology, Hubei University of Science and Technology, Xianning, Hubei Province, China; 2 Hubei Key Laboratory of Diabetes and Angiopathy, School of Pharmacy, Xianning Medical College, Hubei University of Science and Technology, Xianning, Hubei Province, China

**Keywords:** Breast cancer, Ferroptosis, Risk model, Survival risk marker gene, Prognostic tool

## Abstract

This study developed a polygenic risk model for breast cancer (BRCA) prognosis using gene expression and clinical data from the Cancer Genome Atlas (TCGA) and Gene Expression Omnibus (GEO) databases. Differentially expressed genes (DEGs) between tumor and normal tissues were identified using the “limma” R package, yielding 1,000 DEGs (396 upregulated, 604 downregulated). Weighted gene co-expression network analysis (WGCNA) and Lasso-Cox regression integrated ferroptosis- and iron metabolism-related genes, identifying eight key survival risk markers: *ANO6*, *DPYD*, *MMD*, *EGR1*, *ACSL4*, *LIFR*, *PPARG*, and *CREB5*. High-risk groups exhibited elevated *ANO6*, *DPYD*, and *MMD* expression, while *EGR1*, *ACSL4*, *LIFR*, *PPARG*, and *CREB5* showed lower levels. Survival analysis confirmed that low *ANO6* and high *LIFR*/*PPARG* expression correlated with prolonged survival, validated across cohorts. A nomogram incorporating these genes and clinical factors enabled personalized survival probability assessment. The eight-gene model independently stratified high- and low-risk BRCA patients, offering a reliable prognostic tool.

## Introduction

Breast cancer is characterized by a high incidence rate and considerable heterogeneity, constituting one of the leading causes of cancer-related mortality among women globally (1). Epidemiological studies indicate a continuous global increase in breast cancer incidence, with this trend being particularly pronounced in economically developed nations (2). The heterogeneity of breast cancer manifests not only among different patients (inter-tumor heterogeneity) but also within individual tumors (intratumoral heterogeneity) (3). This heterogeneity poses significant challenges for diagnosis and treatment, as various tumor subtypes exhibit differential responses to therapeutic interventions (4). For example, triple-negative breast cancer (TNBC) is often associated with a poor prognosis and reduced survival due to the absence of targeted therapy receptors (5). Furthermore, the pronounced heterogeneity in HER2-positive breast cancer is similarly linked to an unfavorable prognosis (6). The clinical management and prognostic assessment of breast cancer predominantly depend on the TNM classification, clinical staging, hormone receptor status, and HER2 expression levels of the tumor. Nonetheless, the inherent heterogeneity and complexity of breast cancer render these conventional markers occasionally inadequate for accurately predicting patient outcomes or guiding personalized therapeutic strategies. Consequently, the identification of novel prognostic biomarkers has emerged as a pivotal area of research (7,8).

Ferroptosis, a recently characterized form of cell death, has garnered increasing attention for its potential implications in breast cancer. This process, characterized by iron-dependent lipid peroxidation, is intricately linked to the pathogenesis and progression of various malignancies, including breast cancer (9). In the context of breast cancer, dysregulated iron metabolism and the mechanisms governing ferroptosis offer novel insights for therapeutic intervention. Research indicates that the expression of long-chain acyl-CoA synthase 4 (ACSL4) is markedly elevated in breast cancer tissues compared to normal tissues and exhibits a positive correlation with the p53 tumor suppressor factor. This suggests that ACSL4 may contribute to breast cancer pathogenesis by modulating lipid metabolism and ferroptosis pathways (10). Furthermore, the initiation of ferroptosis is intricately linked to alterations in intracellular iron levels. Studies have demonstrated that erastin induces autophagic cell death in breast cancer cells by elevating intracellular iron concentrations, thereby underscoring the pivotal role of iron metabolism in ferroptosis (11). The regulatory mechanisms governing ferroptosis hold significant potential for therapeutic applications in breast cancer treatment. Notably, the GSK3β/Nrf2 signaling pathway has been identified as a critical regulator of erastin-induced ferroptosis. Overexpression of GSK3β enhances erastin-induced ferroptosis, whereas activation of Nrf2 mitigates this effect, highlighting the potential to augment ferroptosis through modulation of the GSK3β/Nrf2 axis (12). Furthermore, the synergistic interaction between NR5A2 and NCOA3 has been shown to mitigate ferroptosis by upregulating NRF2, consequently contributing to breast cancer resistance to BET inhibitors. This discovery offers a novel therapeutic strategy for breast cancer treatment (13). The significance of genes associated with ferroptosis and iron metabolism in breast cancer is increasingly recognized, presenting new avenues for therapeutic intervention. By undertaking comprehensive research into the regulatory mechanisms of ferroptosis and its specific implications in breast cancer, we can establish a theoretical foundation and practical guidance for developing more effective treatment modalities.

In this study, we aimed to utilize public datasets of breast cancer (BRCA) from the the Cancer Genome Atlas (TCGA) and Gene Expression Omnibus (GEO) databases to construct a polygenic risk scoring model through analyses such as Gene Ontology (GO), Kyoto Encyclopedia of Genes and Genomes (KEGG), Gene Set Enrichment Analysis (GSEA), weighted gene co-expression network analysis (WGCNA), and least absolute shrinkage and selection operator (LASSO). Additionally, we assessed the correlation between these BRCA survival risk marker genes and immune cell infiltration, as well as overall survival (OS). We anticipated that this study would provide reliable survival risk marker genes and effective disease testing models, thereby contributing to future scientific research and clinical precision medicine.

## Material and Methods

The research methodology is outlined in the flowchart presented in Supplementary Figure S1.

### Data sources

The primary data source utilized in this study was the BRCA microarray dataset, obtained from the publicly accessible database, Seq TCGA - RNA transcription group (available at https://portal.gdc.cancer.gov/analysis_page?app=Projects). This dataset was generated using the Illumina platform and comprises RNA-seq expression data alongside corresponding clinical information for BRCA patients, all sourced from the Cancer Genome Atlas (TCGA). This integrated dataset served as the training cohort, encompassing 1,098 BRCA samples and 113 normal samples.

For validation purposes, the GSE162228 dataset from the Comprehensive Gene Expression Database (GEO) was employed, which includes 134 BRCA samples. This dataset compared the consistency of oligonucleotide microarray and NanoString nCounter detection platforms in performing PAM50 molecular typing in Taiwanese breast cancer patients. Through the analysis of 109 samples, it was found that there were fundamental differences between the two gene expression measurement methods. The results indicated that the results obtained from different platforms should not be overly emphasized as directly equivalent or mutually substitutable (14).

Furthermore, a total of 839 genes associated with iron metabolism and ferroptosis (collectively termed iron metabolism-related genes) were curated. These genes were identified from 39 iron metabolism pathways obtained from the MSigDB database (accessible at https://www.gsea-msigdb.org/gsea/msigdb/index.jsp) and the ferroptosis gene set from the Ferrdb V2 database.

### Data sets and partitioning

The TCGA-BRCA dataset (n=1,098) was used as the training cohort to construct and fit the prognostic model; meanwhile, the completely independent GEO dataset GSE162228 (n=134) was used as the external validation cohort. The GSE162228 dataset contains samples profiled by different gene expression platforms (oligonucleotide microarray and NanoString nCounter). As the original study has demonstrated that expression values between these platforms may not be directly comparable (14), we treated this external validation as an exploratory analysis. To ensure data quality, we cross-checked the PAM50 classification consistency and retained samples with unambiguous subtype assignments (n=109). Due to the potential batch effects introduced by platform heterogeneity, the validation results from GSE162228 should be interpreted as supportive rather than conclusive evidence.

### Identification of differentially expressed genes (DEGs)

The RNA-seq expression data were normalized utilizing the R package “limma” to mitigate batch effects. This study conducted an analysis to identify and screen DEGs between breast tumor tissues and normal tissues, adhering to the criteria of adjusted P-value <0.01 and |log2FC|>1. The Benjamini-Hochberg (BH) procedure was applied to adjust raw P-values for multiple testing, yielding the adjusted P-values. Visualization was performed using the ggplot2 R package, which facilitated the creation of volcano plots and heat maps. The heat maps were generated by selecting the top 20 up-regulated and top 20 down-regulated genes.

### WGCNA co-expression modules

Based on the identified 5,845 DEGs, we performed WGCNA to transform gene expression data into co-expression gene modules and examine the associations between these modules and clinical characteristics. Initially, the co-expression module was developed utilizing the expression data of DEGs. Raw RNA-seq count data from TCGA-BRCA were processed with the limma-voom pipeline to obtain log2-counts per million (logCPM) values with associated precision weights, which corrects for technical variation across samples without requiring explicit batch covariates. The “remove batch effect” function from the “limma” package in R software was employed to mitigate batch effects in the data. Subsequently, the median absolute deviation (MAD) for each gene was calculated from the gene expression profile. The top 50% of genes with the lowest MAD values were excluded, and the “goodSamplesGenes” function from the WGCNA package in R was applied to remove aberrant genes and samples. The scale independence and average connectivity of the network were assessed across various power values (ranging from 1 to 20). An appropriate power value was selected when the scale independence exceeded 0.89 and the correlation was relatively high. Genes were then categorized into distinct gene modules based on differences in the topological overlap matrix (TOM). Further analysis was conducted to explore the correlation between key gene modules and clinical characteristics. The module showing the highest absolute Pearson correlation with the trait of interest (tumor *vs* normal status) in the module-trait relationship heatmap was designated as the key module associated with breast cancer. The ‘brown’ module met this criterion and was selected for downstream analysis. The module-trait heatmap and dendrogram are displayed in Supplementary Figure S2C and D, confirming the brown module's strongest correlation with tumor status.

### Construction and evaluation of prognostic models for BRCA iron metabolism-related genes

In this study, the brown module exhibiting the highest co-expression correlation with BRCA, as constructed by WGCNA, was intersected with the DEGs from BRCA-RNA-Seq, as well as genes associated with ferroptosis and iron metabolism. A predictive model was developed using the LASSO Cox regression method. To mitigate the risk of overfitting, the “glmnet” function from the R package was utilized. Survival time, survival status, and gene expression data were integrated, and regression analysis was performed using the LASSO-Cox approach. Furthermore, a 10-fold cross-validation was implemented to identify the optimal model. To obtain a more parsimonious and stable prognostic signature, the lambda value corresponding to the lambda.1se criterion (λ=0.0125) was selected, resulting in the identification of eight genes with non-zero coefficients.

Subsequently, patient risk score was calculated based on gene expression levels and the corresponding Cox regression coefficients, as follows: RS = Σ^n^_i=1_ (β_i_×Exp_i_) = (−0.0203×Exp_PPARG_)+(−0.0319×Exp_LIFR_)+ (0.0062×Exp_MMD_)+(−0.1054×Exp_CREB5_)+(−5.7225×10^−5^×Exp_EGR1_)+ (0.0315×Exp_ANO6_)+(−1.4767×10^−4^×Exp_DPYD_)+(−0.0046×Exp_ACSL4_), where βi represents the LASSO-Cox regression coefficient of gene i, and Expi represents the expression level of this gene (after standardization).

Subsequently, the cohort was divided into high-risk group and low-risk group based on the median of all patients' risk scores. To preliminarily assess whether the risk score was independent of intrinsic breast cancer subtypes, the distribution of PAM50 molecular subtypes (luminal A, luminal B, HER2-enriched, basal-like, Normal-like) within the high- and low-risk groups in both the TCGA training set and the GSE162228 validation set was examined.

### Survival analysis

Patients were dichotomized into high- and low-risk groups by the median risk score. Kaplan-Meier survival curves were generated to visualize OS, and the log-rank test was used to assess the significance of survival differences between the two groups. A forest plot was subsequently generated using the R package “Forest Plot” to illustrate the hazard ratio (HR), 95% confidence interval (CI), and P-value. Furthermore, a nomogram was developed utilizing the R software package “rms”. Additionally, survival analysis of key genes was performed using the kmplot platform (https://kmplot.com). In the TCGA training cohort, the median follow-up time was 42 months (range: 3-81 months). The GSE162228 dataset provides complete time-to-event (OS in months) and censoring status for each patient, enabling standard Kaplan-Meier and Cox regression analyses. The earlier statement that follow-up was not provided was a misinterpretation. All survival analyses (OS) were based on the events (deaths) recorded in the complete GEO clinical records.

Univariate and multivariate Cox proportional hazards regression models were applied to evaluate the prognostic significance of the 8-gene risk score and clinical covariates (including age, gender, stage, and TNM classification). The risk score was treated as a continuous variable in primary inference.

### Protein-protein interaction network and functional enrichment analysis

To investigate the interactions between the central gene and other associated genes, the relevant genes were analyzed using the Search Tool for the Retrieval of Interacting Genes/Proteins (STRING) database (https://string-db.org/), with a minimum confidence threshold set at >0.4. This online platform provides an extensive repository of integrated and consolidated protein-protein interaction data. The protein-protein interaction (PPI) network was constructed utilizing Cytoscape 3.8.0 software, followed by further enrichment analysis of these genes. Subsequently, the KEGG pathway analysis was performed using the “clusterProfiler” R package, with P-values adjusted according to the Benjamini and Hochberg method. GO functional enrichment analysis was conducted on the top 100 co-expressed genes, focusing on cellular components (CC) and biological processes (BP). Additionally, annotations were made for molecular functions (MF).

### Analysis of immune cell infiltration levels

To investigate the expression of hub genes and the infiltration of 21 types of immune cells, we utilized the TIMER2.0 database (http://www.timer.cistrome.org/), a widely recognized resource for studying tumor-immune cell interactions. The infiltration levels of immune cells were assessed using the Tumor Immune Estimation Resource (TIMER) methodology. The immune cell types analyzed included naive B cells, monocytes, memory B cells, M0 macrophages, plasma cells, M1 macrophages, CD8 T cells, M2 macrophages, resting CD4 memory T cells, resting dendritic cells, activated CD4 memory T cells, activated dendritic cells, follicular helper T cells, resting mast cells, regulatory T cells (Tregs), activated mast cells, gamma delta T cells, eosinophils, resting natural killer (NK) cells, neutrophils, and activated NK cells. The analysis was adjusted for tumor purity. Furthermore, BRCA patients were stratified into two groups based on the expression levels of the hub gene to explore the correlation between the hub gene expression and the immune microenvironment scores of the 21 immune cell types.

### Gene set enrichment analysis

GSEA was performed to identify KEGG pathways significantly enriched in association with the high-risk phenotype. A pre-ranked gene list was generated based on the moderated *t*-statistic (log2 fold change) derived from comparing the high- and low-risk groups. GSEA was then conducted using the clusterProfiler package (version 4.5) against the c2.cp.kegg.v7.4.symbols.gmt gene set collection. The ranking metric used was the log2 fold change in expression between the high-risk and low-risk groups. To ensure a robust signal, the analysis focused on samples with extreme risk scores (top 30% *vs* bottom 30% of the risk score distribution). Each analysis involved 1,000 gene set permutations. Gene set enrichment significance was assessed based on two metrics: a nominal P-value (unadjusted for multiple comparisons) <0.05 and a false discovery rate (FDR) *q*-value <0.25. The FDR threshold of 0.25 is in accordance with the widely adopted standard for exploratory GSEA, which aims to balance the sensitivity for discovering biologically relevant pathways with an acceptable level of false positives.

For the expression analysis of BRCA survival risk marker genes related to ferroptosis, the Wilcoxon rank-sum test was applied for the comparison of hub gene expression levels between tumor and normal tissues in the TCGA cohort. Data were visualized using the “ggplot2” R package, and a P-value <0.05 was considered statistically significant.

### Statistical analysis

All statistical analyses and the calculation of inferential P-values were performed using R software (v4.0.2). IBM SPSS 21.0 (IBM SPSS, USA) was occasionally used for data preprocessing and descriptive statistics for compatibility with certain visualization packages. Visualization of results, including differential gene expression, immune infiltration, and survival analysis, was conducted utilizing the networkD3 R software package. The Kaplan-Meier survival curve was analyzed using the log-rank test. For comparisons between two groups, the Mann-Whitney U test was applied, while the Kruskal-Wallis test was utilized for comparisons among multiple groups. When the omnibus test was significant, Dunn's multiple comparison test with Benjamini-Hochberg adjustment was performed for pairwise comparisons. The Wilcoxon test was utilized for comparisons among multiple groups. The Pearson correlation coefficient was used to assess the linear relationship between quantitative variables. All statistical P-values (or adjusted P-values) were computed using R software (v4.0.2), with a P-value of less than 0.05 considered statistically significant. For analyses involving multiple simultaneous comparisons (e.g., correlation between hub gene expression and immune cell infiltrations across 21 cell types), Benjamini-Hochberg adjusted P-values (FDR) are reported alongside nominal P-values. Results with FDR<0.05 were considered significant after correction; uncorrected P*-*values are explicitly noted and interpreted with caution for hypothesis generation only.

## Results

### BRCA differential expression analysis

Principal component analysis (PCA) was employed to assess the repeatability of data within both the control and BRCA sample groups. Following this, the TCGA-biliary tract cancer dataset underwent standardization. Utilizing significance criteria (P-value <0.01) and fold-change thresholds (logFC≥1), a total of 5,845 DEGs were identified from the BRCA dataset. This included 2,140 up-regulated genes and 3,705 down-regulated genes, as detailed in Supplementary Table S1, which shows only the 50 genes with the most significant upregulation and downregulation (for detailed information, please contact the corresponding author). The distribution of these DEGs is illustrated in the volcano plot (Figure 1A). Furthermore, a heat map depicting the expression profiles of the top 20 DEGs was constructed (Figure 1B).

**Figure 1:**
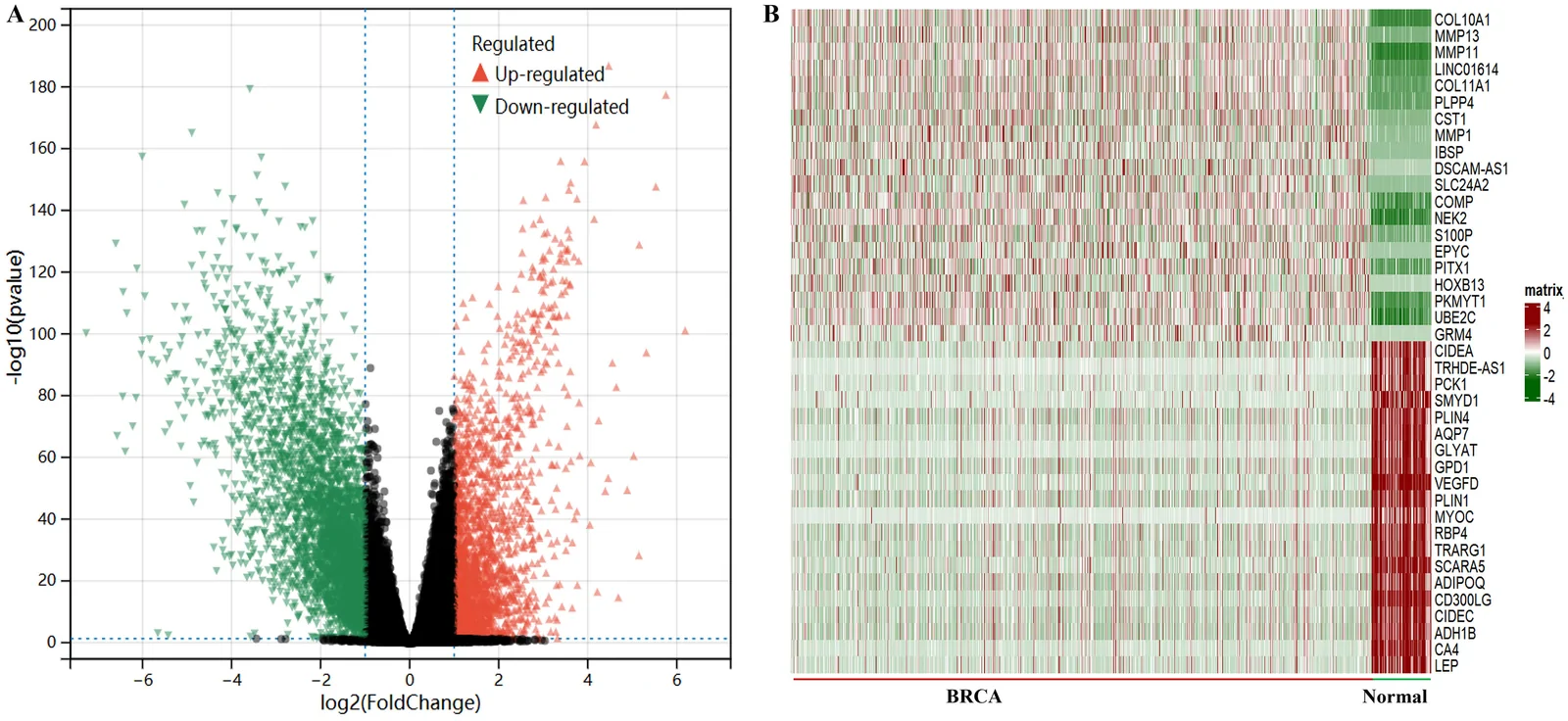
Differentially expressed genes in the Cancer Genome Atlas - Breast Cancer (TCGA-BRCA) database. **A**, Volcano plots depicting differentially expressed genes between the Normal and BRCA groups. **B**, Heat map highlighting the top 20 genes exhibiting significant expression differences.

### Access key modules of the BRCA transcriptome

The DEGs identified were analyzed using WGCNA to investigate the relationship between gene modules and specific clinical features. Clinical data for BRCA patients were obtained from the TCGA database. The soft-thresholding power (β) was chosen as 5, where the scale-free topology fit index R^2^ first reached a plateau above 0.89 and mean connectivity remained sufficiently high to maintain a biologically meaningful network (Supplementary Figure S2A and B). This satisfied the scale-free network assumption. Typically, when the fitting coefficient R^2^ approaches or reaches 0.9, the optimal soft threshold β is determined as the minimum soft threshold. Utilizing dynamic tree cutting with a cutting height of 0.2 and a minimum module size of 50, a dendrogram of gene clusters was produced, resulting in the identification of eight co-expression modules (Supplementary Figure S2C). The Pearson correlation method was employed to assess the relationship between each module and the clinical data of BRCA patients, with the brown module exhibiting the strongest correlation with clinical features (Supplementary Figure S2D). A heat map was generated to visualize the correlation distances among characteristic genes (Supplementary Figure S2E). The relationship between module membership (MM) and gene significance (GS) was evaluated (R=0.92) (Supplementary Figure S2F).

### Screening of core genes associated with iron metabolism in BRCA and the development and validation of prognostic models

In this study, we identified DEGs from a dataset of 5,845 genes associated with BRCA. We conducted an analysis to determine the intersection of genes from the brown module (comprising 651 genes) and those related to iron metabolism (comprising 839 genes) using a weighted gene co-expression network analysis (Figure 2A). We conducted an analysis to determine the intersection of genes from the brown module (comprising 651 genes) and those related to iron metabolism (comprising 839 genes), and this analysis yielded 31 intersection genes, which we designated as the core genes involved in iron metabolism in BRCA (Table S2). Utilizing comprehensive data on survival time, survival status, and gene expression, we applied Lasso-Cox regression analysis to these 31 core genes. At the optimal tuning parameter (λ) of 0.0125, we identified eight significant biomarkers: *ANO6*, *MMD*, *EGR1*, *ACSL4*, *DPYD*, *LIFR*, *PPARG*, and *CREB5* (as shown in Figure 2B and C). Subsequently, we developed an 8-gene prognostic model to predict patient outcomes. In the TCGA cohort, patients were stratified into high-risk and low-risk groups based on the median risk score. The prognostic model is represented by the following equation: RiskScore = -0.0202587287285268×PPARG-0.0318605733215718×LIFR+0.00620178824575923×MMD-0.105403553646483×CREB5-5.72251181874526e-05×EGR1+0.0315401475914519×ANO6-0.000147665163517008×DPYD-0.00460299804576539×ACSL4.

**Figure 2:**
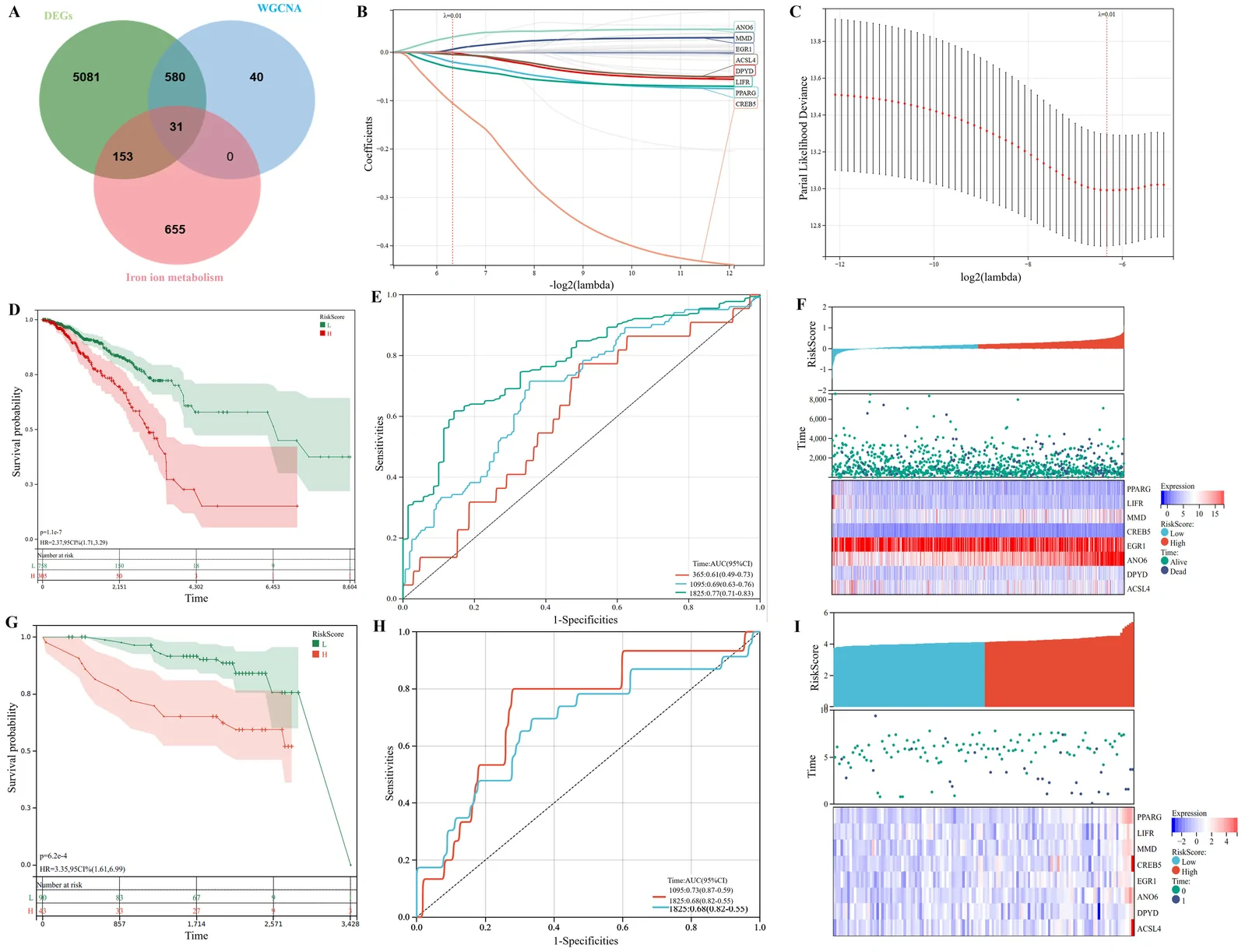
Development and assessment of prognostic models for genes associated with iron metabolism. **A**, The Venn diagram illustrates the overlap among differentially expressed genes (DEGs), genes from the brown module, and genes related to ferroptosis and metabolism. **B**, LASSO regression analysis was conducted to identify the optimal hub gene within the Lasso model (λ). **C**, The coefficient distribution graph of the logarithmic (λ) sequence was generated through LASSO regression analysis. **D**, Survival analysis of the prognostic models was performed. **E**, Receiver operating characteristic (ROC) curve of the prognostic model. **F**, Distribution of risk scores within the prognostic model, risk score density distribution, and Z-correction values in the heat maps for the expression of eight genes. **G**, Validation of the survival analysis for the model. **H**, Validation of the ROC curve for the model. **I**, Validation of the risk score distribution for the model, including the risk score density distribution and Z-correction values in the heat maps for the expression of the eight genes. H: high risk; L: low risk.

In the training cohort model, survival analysis demonstrated that the risk score significantly influenced the prognosis of BRCA patients (P<1.1e-7) (reported P-values are from the log-rank test), with a lower survival rate observed in the high-risk score group (Figure 2D). The ROC analysis curve indicated that the AUC at 365 days was 0.61 (95%CI: 0.49-0.73), at 1095 days was 0.69 (95%CI: 0.63-0.76), and at 1825 days was 0.77 (95%CI: 0.71-0.83) (Figure 2E), suggesting that the model had good predictive capability. The association between the prognostic model and gene expression in the training cohort is depicted in Figure 2F. The predictive model was subsequently validated using the GSE162228 dataset, where similar results were observed, showing a lower survival rate in the high-risk score group with a significant difference (P<6.2e-4) (Figure 2G). The AUC at 1,095 days was 0.68 (95%CI: 0.87-0.59), and at 1,825 days, it was 0.68 (95%CI: 0.82-0.55) (Figure 2H). The relationship between the prognostic model and gene expression is illustrated in Figure 2I. Overall, these findings suggest that the established prediction model had strong predictive performance.

### Cox regression analysis of 8-gene marker characteristics

To elucidate the prognostic significance of the 8-gene marker in patients with BRCA, we performed a subgroup survival analysis focusing on risk scores associated with independent prognostic factors, specifically the M stage and clinical stage. The samples were stratified into distinct cohorts based on these stages, and the prognostic utility of the BRCA risk score was assessed using Kaplan-Meier analysis. The findings indicated that, even after adjusting for confounding variables such as M stage and clinical stage, a significant difference persisted between the high-risk and low-risk groups when characterized by the 8-gene signature (P<0.05) (Figure 3A-D).

**Figure 3:**
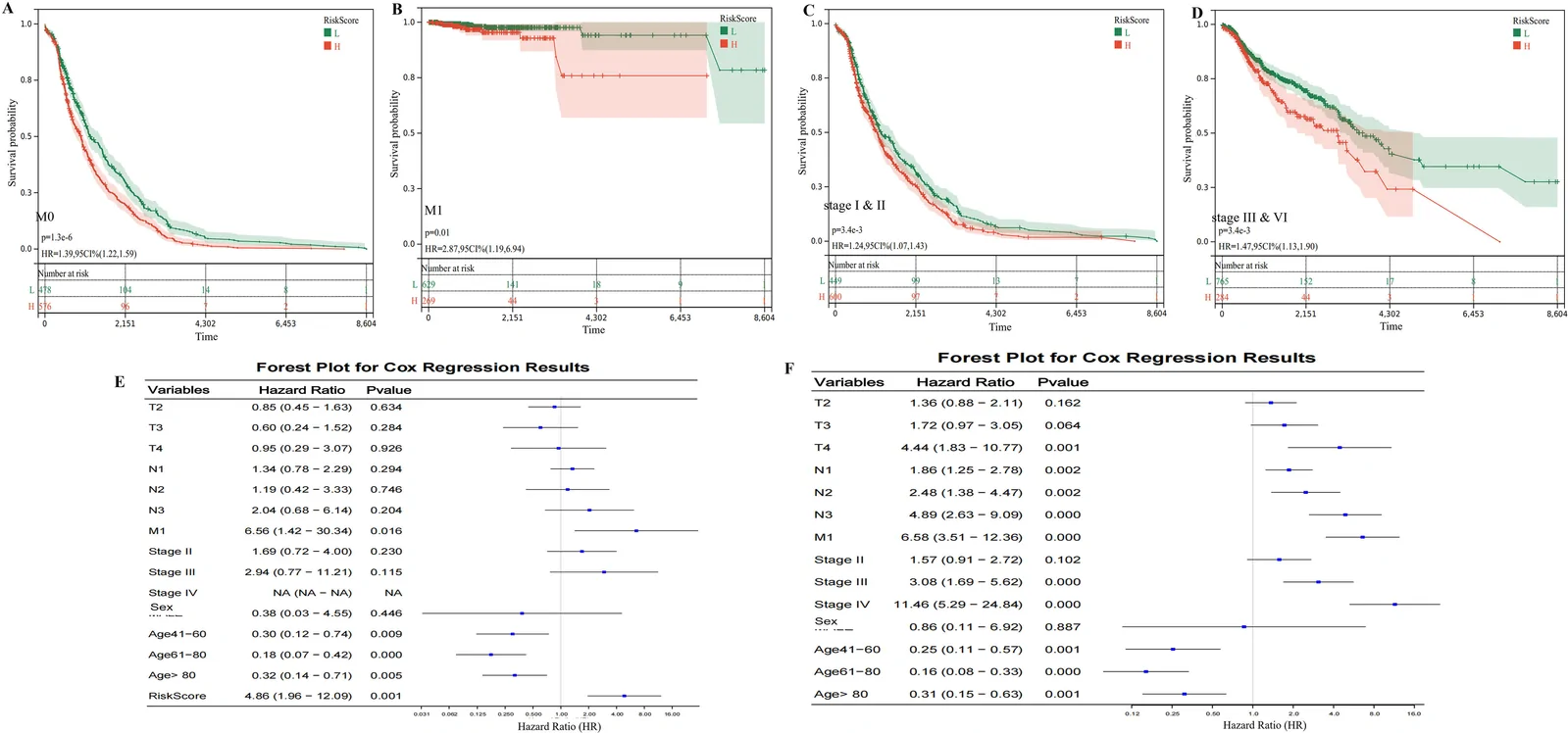
Univariate and multivariate Cox regression analyses of gene characteristics and subgroup survival analyses. **A**-**D**, Survival analyses across different subgroups were conducted to evaluate the independence of risk scores (H: high risk; L: low risk). **E**, Forest plot of the multivariate Cox model is provided. **F**, Forest plot derived from the univariate Cox proportional hazards model. The hazard ratio (HR) serves as a relative measure for assessing the prognosis of breast cancer patients.

Further, Cox regression analyses were applied to the risk scores derived from the eight genes, alongside variables such as age, gender, clinical stage, and the pathological tumor, lymph node, and metastasis (T, N, M) stages. In multivariate analysis, the 8-gene risk score, M stage, and age groups emerged as independent predictors of patient prognosis (Figure 3E; P<0.05), whereas no statistically significant differences were observed for T, N, clinical stage, and gender. In the univariate analysis, the 8-gene risk score, T4, N, M stage, clinical stage, and various age groups demonstrated a significant association with prognosis (Figure 3F). Conversely, no statistically significant differences were observed in T2, T3, and gender (P<0.05).

### PPI network analysis and gene enrichment analysis

Utilizing the 8 BRCA survival risk marker genes associated with ferroptosis identified previously, the “STRING” database facilitated the construction of a PPI network between these 8 BRCA survival risk marker genes and other related genes. The PPI network indicated that certain proteins, such as JAK2, IL6, JUN, GPX4, and NCOR1, exhibit a strong association with the BRCA survival risk marker genes linked to ferroptosis (Figure 4A). Furthermore, KEGG and GO enrichment analyses were performed on the aforementioned 8 BRCA survival risk marker genes related to ferroptosis. The results of the KEGG enrichment analysis revealed that these genes are involved in several pathways, including the PI3K-Akt signaling pathway, TNF signaling pathway, estrogen signaling pathway, Jak-STAT signaling pathway, human cytomegalovirus infection, glucagon signaling pathway, insulin resistance, AMPK signaling pathway, and IL-17 signaling pathway (Figure 4B). The GO enrichment analysis indicated that, within the BP category, the genes were primarily associated with cell differentiation, positive regulation of developmental processes, positive regulation of transcription by RNA polymerase II, response to cytokines, hormones, lipids, organic cyclic compounds, steroid hormones, and circadian rhythm (Figure 4C). In the CC category, the genes were mainly linked to the chromosome, transcription factor complex, RNA polymerase II transcription factor complex, and transcription factor AP-1 complex (Figure 4D). Within the MF category, the genes are predominantly involved in signaling receptor binding, transcription factor binding, transcription regulatory region DNA binding, regulatory region nucleic acid binding, DNA-binding transcription activator activity specific to RNA polymerase II, hormone receptor binding, nuclear hormone receptor binding, activating transcription factor binding, RNA polymerase Il activating transcription factor binding, and cAMP response element binding (Figure 4E).

**Figure 4:**
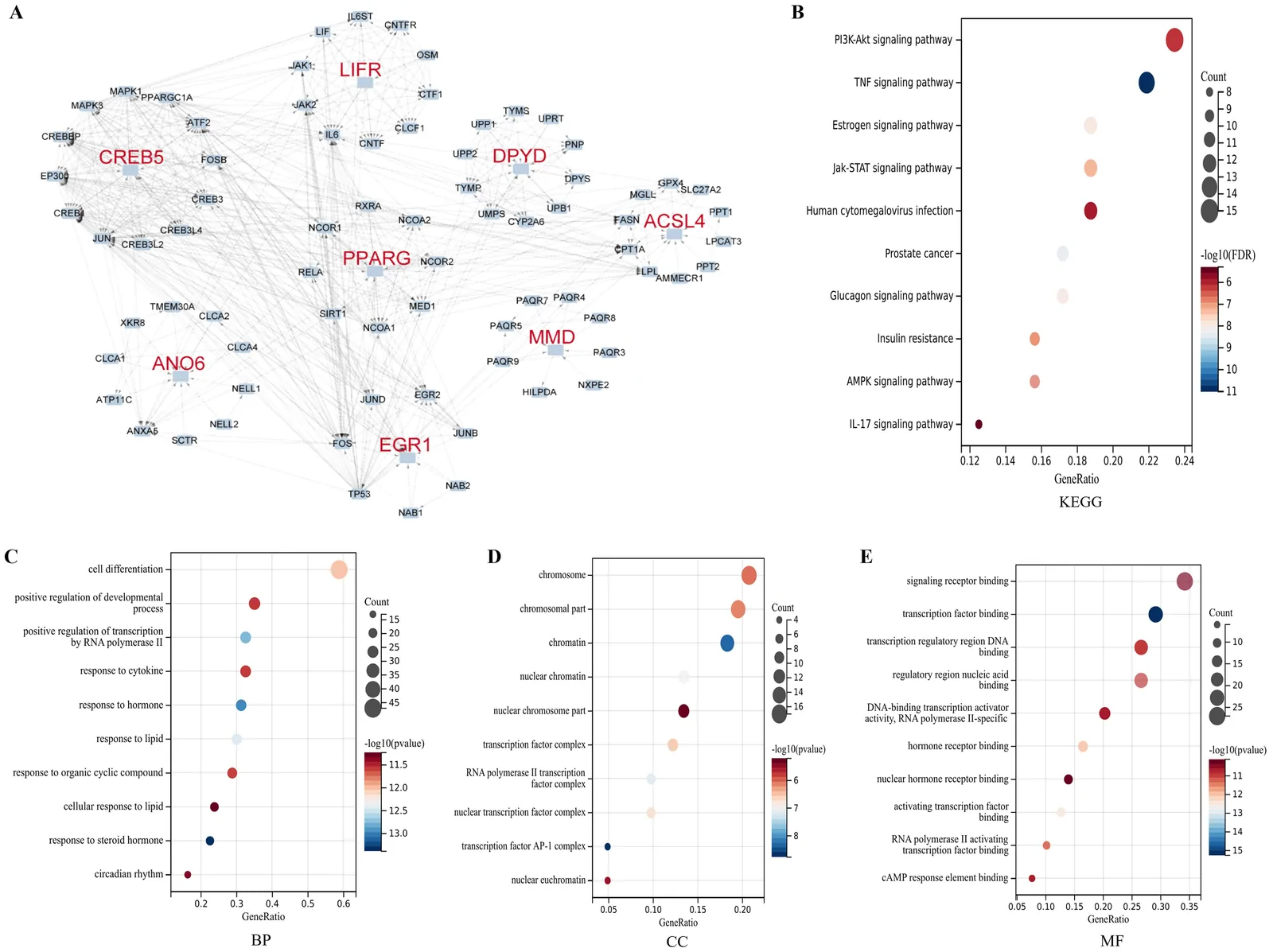
Construction of protein-protein interaction (PPI) networks and the enrichment analysis of associated genes. **A**, Identification of eight hub genes within the comprehensive PPI network, where red dots denote hub genes and blue dots represent associated genes. **B**, KEGG pathway enrichment maps for the eight hub genes and their associated genes. **C**, Gene Ontology (GO) enrichment analysis of biological processes (BP), **D**, cellular components (CC), and **E**, molecular functions (MF) for the eight hub genes and their associated genes in breast cancer.

### Training of the cohort model and survival risk marker genes with respect to cellular immune infiltration

The tumor microenvironment primarily consists of tumor-associated fibroblasts, immune cells, extracellular matrix, various growth factors, inflammatory mediators, distinct physicochemical properties, and the cancer cells themselves. This microenvironment plays a crucial role in influencing the diagnosis, survival, and treatment responsiveness of malignant tumors. By examining the relationship between the training cohort model and tumor immune infiltration, we further investigated the potential molecular mechanisms by which key genes impact the progression of BRCA. In this analysis, we assessed various immune cell types, including naive B cells, monocytes, memory B cells, M0 macrophages, plasma cells, M1 macrophages, and CD8 T cells, in both high-risk and low-risk patients within the training cohort model. Additionally, we examined M2 macrophages, resting and activated CD4 memory T cells, resting and activated dendritic cells, follicular helper T cells, resting and activated mast cells, Tregs, gamma delta T cells, eosinophils, resting NK cells, neutrophils, and activated NK cells.

Supplementary Figure S3A and B presents the heat maps and cell ratios of immune-infiltrating cells from high-risk and low-risk patients within the training cohort model. In comparison to the low-risk group, the high-risk group exhibited a significant increase in the proportions of immune-infiltrating cells, including resting NK cells, M0 macrophages, resting mast cells, and neutrophils. Conversely, there was a significant decrease in the proportions of CD8 T cells, activated CD4 memory T cells, activated NK cells, and resting dendritic cells (Supplementary Figure S3C; P<0.05).

The TIMER database was utilized to investigate the immune microenvironment, specifically examining the correlation between immune infiltration levels in BRCA and the expression of survival risk marker genes associated with ferroptosis (Supplementary Figure S3D). The findings revealed that *PPARG* expression exhibited a positive correlation with resting dendritic cells and a negative correlation with neutrophils, follicular helper T cells, and Tregs. Similarly, MMD expression was positively correlated with M1 macrophages, activated CD4 memory T cells, and gamma delta T cells, while showing a negative correlation with monocytes. LIFR expression demonstrated a positive correlation with CD8 T cells and a negative correlation with M0 macrophages, neutrophils, follicular helper T cells, and Tregs. EGR1 expression was positively correlated with naive B cells, activated mast cells, and resting CD4 memory T cells, and negatively correlated with Tregs. Lastly, CREB5 expression was positively correlated with naive B cells and activated mast cells, but negatively correlated with resting mast cells, neutrophils, and Tregs. The expression of ANO6 exhibited a positive correlation with M0 macrophages and resting mast cells, while demonstrating a negative correlation with activated NK cells and CD8 T cells. Conversely, ACSL4 expression was positively correlated with M1 macrophages, activated memory CD4 T cells, CD8 T cells, eosinophils, M2 macrophages, and resting mast cells (Supplementary Figure S3D).

### Analysis of differential genes and GSEA enrichment in high- and low-risk groups

Differential gene expression analysis was performed on the high-risk and low-risk groups within the training cohort model. The findings indicated that, in comparison to the low-risk group, the high-risk group exhibited significantly elevated expression levels of BRCA survival risk marker genes associated with ferroptosis, including *ANO6*, *DPYD*, and *MMD*. In contrast, the expression levels of genes such as *EGR1*, *ACSL4*, *LIFR*, *PPARG*, and *CREB5* were significantly reduced (Figure 5A and B).

**Figure 5:**
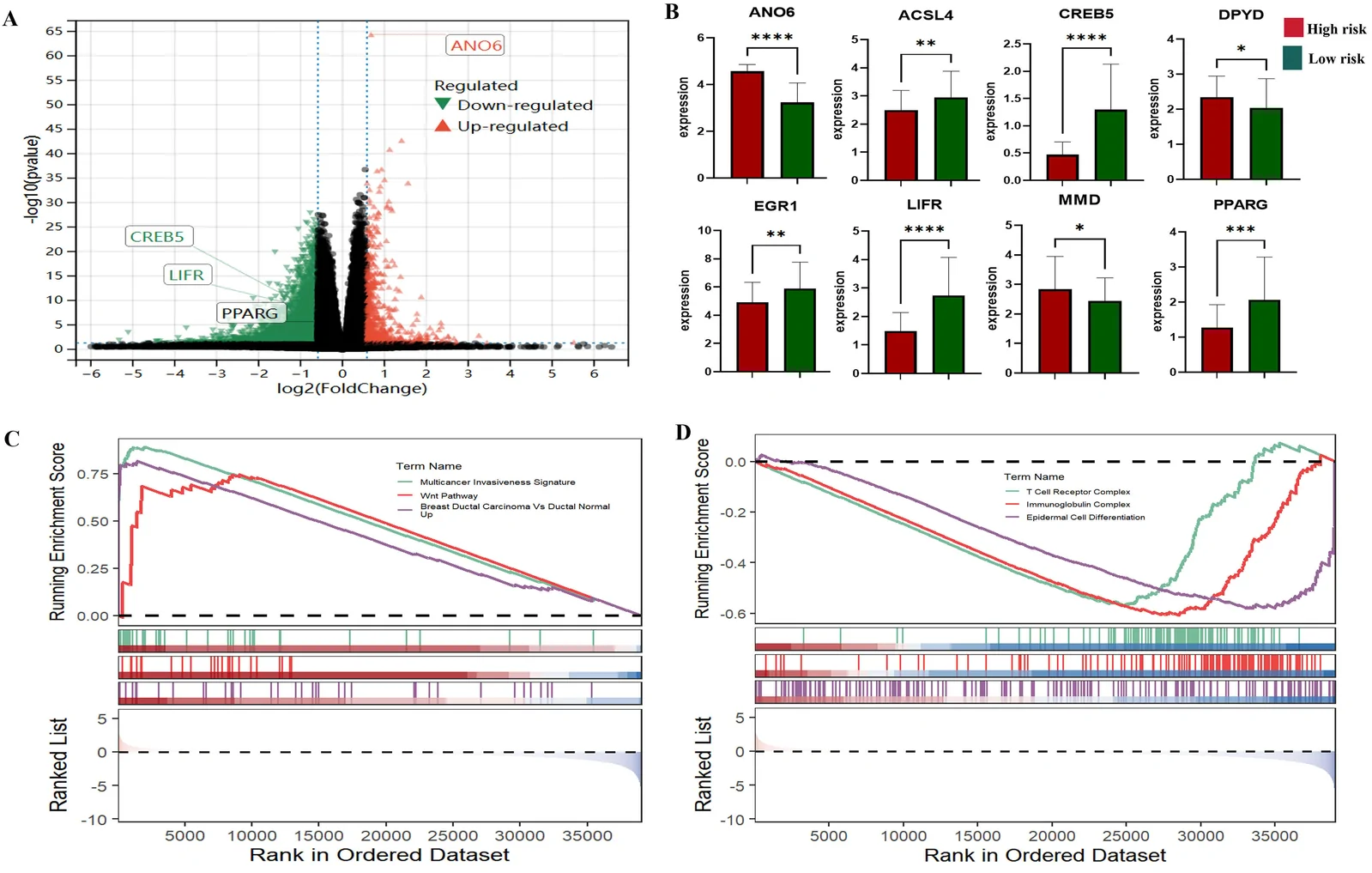
Enrichment analysis of differential genes and Gene Set Enrichment Analysis (GSEA) in the high-risk and low-risk groups of breast cancer (BRCA). **A** and **B**, analysis of differential genes between these groups, highlighting the expression levels of the eight hub genes. **C** and **D**, GSEA results for the high-risk group. Data are reported as mean and SD. *P<0.05, **P<0.01, ***P<0.001, ****P<0.0001. Student's *t*-test.

GSEA revealed that in the high-risk group, pathways related to cancer invasion, the Wnt signaling pathway, and upregulation of ductal carcinoma of the breast were activated. Meanwhile, pathways involving the T-cell receptor complex, immunoglobulin complex, and endothelial cell differentiation were inhibited (Figures 5C and D).

### Construction of monograms

The construction of monograms facilitated the development of a survival prediction map capable of integrating various clinical prognostic factors to assess survival probabilities at 1, 3, and 5 years. Consequently, a risk prediction nomogram was developed, incorporating risk coefficients, TN stage, age, stage, and gender (Figure 6). This model demonstrated a C-index of 0.747, a hazard ratio ranging from 0.448 to 0.945, and a P-value of 2.33e-11. These findings indicate that the model effectively determined disease progression in patients and provided personalized predictions for individuals with BRCA.

**Figure 6:**
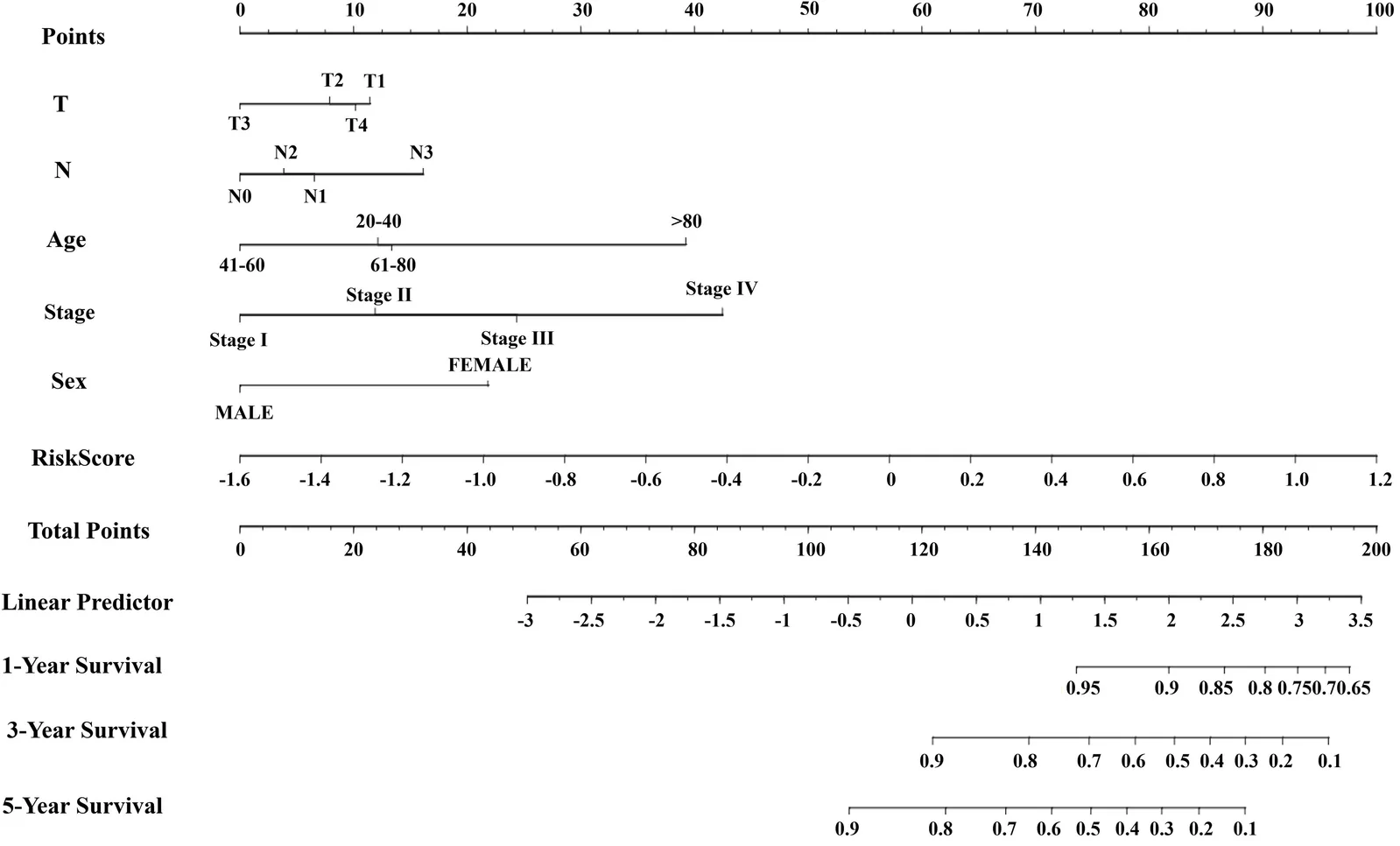
Construction of a nomogram designed to predict survival of breast cancer patients by integrating various clinical prognostic factors, thereby estimating survival probabilities at 1, 3, and 5 years. This risk prediction nomogram incorporates risk coefficient, T and N stages, age, stage, and gender.

### Survival validation of the expression of BRCA survival risk marker genes related to ferroptosis

In the TCGA-BRCA dataset, survival validation of BRCA survival risk marker genes associated with ferroptosis revealed that low expression of *ANO6* was significantly correlated with prolonged survival, whereas high expression of *LIFR* and *PPARG* was significantly associated with extended survival (Figure 7A-H). Conversely, the expressions of *DPYD*, *MMD*, *EGR1*, *ACSL4*, and *CREB5* were not correlated with survival outcomes (Figure 7A-H). Thus, the survival analysis indicated that low expression of *ANO6* and high expression of *LIFR* and *PPARG* were significantly linked to longer survival durations. Furthermore, a systematic analysis was conducted to examine the differences in mRNA expression levels of *ANO6*, *LIFR,* and *PPARG* in various breast cancer subtypes. The Kruskal-Wallis test was used for multiple group comparisons, followed by Dunn's post hoc test with BH adjustment. The results indicated that compared to the HER2-enriched subtype and the triple-negative subtype, the specific genes *ANO6*, *LIFR,* and *PPARG* exhibited significantly higher expression in the luminal subtype (Supplementary Figure S4A-C).

**Figure 7:**
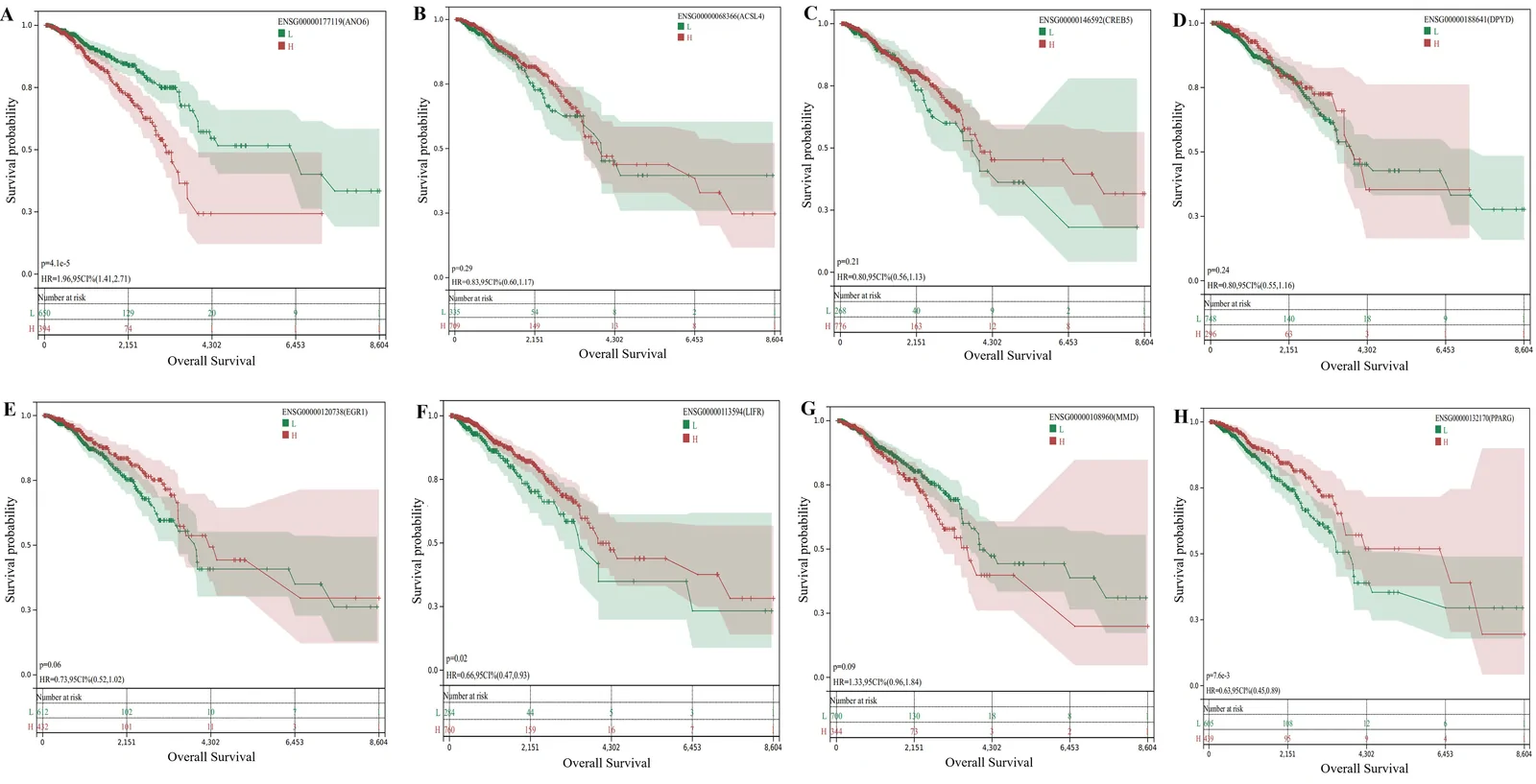
Validation of core gene expression characteristics. **A**-**H**, The Kaplan-Meier curve analysis was employed to assess the relationship between gene expression and prognosis. H: high risk; L: low risk.

## Discussion

Ferroptosis, as a newly discovered form of cell programmed death, plays a significant role in the occurrence and development of various cancers. In recent years, researchers have gradually recognized the potential prognostic value of ferroptosis-related genes in breast cancer (BRCA). Genes such as *ANO6*, *ACSL4*, *CREB5*, *DPYD*, *EGR1*, *LIFR*, *MMD*, and *PPARG* have certain reference significance as prognostic markers for BRCA patients. Firstly, as a key regulatory factor of ferroptosis, *ACSL4* expression in breast cancer is closely related to the prognosis of patients. Studies have shown that when *ACSL4* is highly expressed in breast cancer cells, it can promote the occurrence of ferroptosis, thereby inhibiting tumor growth and metastasis (15). Moreover, *ACSL4* also interacts with other ferroptosis-related genes such as *GPX4*, affecting the response of breast cancer patients to neoadjuvant chemotherapy (15). These research results indicate that *ACSL4* is not only a key regulatory factor of ferroptosis but also an important prognostic factor for breast cancer. Secondly, *EGR1* and *LIFR* in breast cancer have also attracted attention. As a transcription factor, *EGR1* expression in various cancers is related to the invasiveness of tumors and the prognosis of patients (16), while *LIFR* is considered to be related to ferroptosis in gallbladder cancer and has a significant correlation with immune cell infiltration, suggesting its potential role in breast cancer as well (17). Changes in the expression of these genes may affect the prognosis of breast cancer by regulating the immune response in the tumor microenvironment. Furthermore, *ANO6* and *PPARG* also deserve attention in breast cancer. *ANO6* is considered a ferroptosis-related marker in cervical squamous cell carcinoma, and its expression level is significantly correlated with the prognosis of patients (18). Through wound healing experiments and transwell experiments, the effects of *ANO6* overexpression on the invasion and metastasis of breast cancer cells were studied (19). Although PPARG has been studied as a potential therapeutic target in various cancers, its specific mechanism of action in breast cancer still needs further exploration.

Anoctamin 6 (*ANO6*), a member of the calcium-activated chloride channel (CaCC) family, is expressed in three splicing variants (V1, V2, and V5) on the plasma membrane (20). The involvement of ANO6 in tumorigenesis and its potential utility as a prognostic biomarker have garnered considerable scholarly interest (21-23). Empirical studies have demonstrated that ANO6 plays a pivotal role in various cancers, with a pronounced impact on breast cancer, where its expression level is closely correlated with patient prognosis. Notably, ANO6 expression is significantly reduced in breast cancer tissues compared to normal breast tissues; however, its overexpression serves as an independent predictor of poor OS in breast cancer patients (24). Furthermore, ANO6 is implicated in modulating immune characteristics within the tumor microenvironment, particularly influencing the expression levels of immune checkpoints, tumor mutational burden, and microsatellite instability (24).

The role of ANO6 within the tumor microenvironment extends beyond its potential as a prognostic marker, encompassing its involvement in ferroptosis and immune cell infiltration. Studies have demonstrated that ANO6 influences the progression of Alzheimer's disease by modulating lipid peroxidation and ferroptosis triggered by endoplasmic reticulum stress (25). In the context of gastrointestinal stromal tumors, ANO6 expression has been shown to inhibit tumor growth and induce ferroptosis, indicating its potential to prevent tumor development through the induction of apoptosis, pyroptosis, and ferroptosis (21). Furthermore, in breast cancer, ANO6 is linked to macrophage polarization, particularly the polarization of M1-type tumor-associated macrophages, which may impact the development of cancer-related anemia (26). Additionally, ANO6 is implicated in matrix-related pathways, with its elevated expression significantly correlated with a high matrix score and macrophage polarization (24). These findings suggest that ANO6 serves not only as an independent prognostic factor for breast cancer but also potentially modulates breast cancer progression through the regulation of matrix-related pathways and macrophage polarization.

The leukemia inhibitory factor receptor (*LIFR*) has garnered considerable interest for its role in various tumors and its potential utility as a prognostic biomarker. LIFR is implicated in significant biological processes in breast cancer and is intricately associated with ferroptosis, iron metabolism, the immune microenvironment, and immune infiltration. Research indicates that LIFR is markedly expressed in TNBC and is significantly correlated with patient OS rates (27). Furthermore, LIFR influences the proliferation and migration of breast cancer cells via its interaction with the JAK/STAT signaling pathway (28). These findings suggest that LIFR could serve as a potential therapeutic target for breast cancer. In the context of gastric cancer, LIFR also exhibits notable biological functions. Studies have demonstrated that LIFR facilitates the proliferation, migration, and invasion of gastric cancer cells through the HIPPO-YAP signaling pathway (29). Furthermore, the interaction between LIFR and the long non-coding RNA (lncRNA) LIFR-AS1 has been substantiated as a critical factor in the progression of gastric cancer. LIFR-AS1 modulates the expression of LIFR through the sponge adsorption of microRNA (miRNA), thereby impacting the biological behavior of cancer cells (30). The involvement of LIFR in ferroptosis and iron metabolism has also been increasingly elucidated. Research indicates that LIFR is intricately linked to the expression of genes related to iron metabolism, potentially influencing tumor cell survival and death by modulating iron metabolic pathways (31). Additionally, the significance of LIFR in the tumor immune microenvironment warrants attention. LIFR affects the immune microenvironment by regulating immune cell infiltration and immune responses, consequently influencing tumor progression and prognosis (32).

The involvement of peroxisome proliferator-activated receptor γ (*PPARG*) in oncogenesis and its potential utility as a prognostic biomarker have garnered significant scholarly interest. PPARG demonstrates diverse functions across different cancer types, exhibiting both tumor-suppressive and tumor-promotive roles depending on the context. Empirical evidence indicates a strong correlation between PPARG expression and patient prognosis in breast cancer, particularly among COX-1-negative cases. Notably, cytoplasmic expression of PPARG is linked to a diminished survival rate, underscoring its prognostic value in breast cancer (33). Furthermore, the influence of PPARG within the immune microenvironment warrants attention. Recent studies have elucidated that PPARG enhances the anti-tumor efficacy of tumor-infiltrating natural killer T (iNKT) cells through the modulation of lipid metabolism. Specifically, PPARG collaborates with PLZF to facilitate cholesterol synthesis, thereby augmenting IFN-γ production in iNKT cells, which is pivotal for bolstering the immune response (34). This mechanism suggests that PPARG plays a critical role in modulating the tumor immune microenvironment and may serve as a potential target for enhancing the efficacy of immunotherapy. Additionally, PPARG is implicated in the regulation of ferroptosis and iron metabolism. Research has demonstrated that PPARG can inhibit aerobic glycolysis (Warburg effect) in hepatocellular carcinoma by modulating the WNT/β-catenin/pyruvate dehydrogenase kinase isoenzyme 1 (PDK1) axis, thereby suppressing tumor metastasis (35). This mechanism not only elucidates the role of PPARG in tumor metabolic regulation but also suggests its potential involvement in regulating the survival and death of tumor cells through the modulation of iron metabolic pathways.

Targeting the ferroptosis pathway may be a potential therapeutic strategy for breast cancer across subtypes, and this view has received increasing support in recent studies (36). Firstly, the mechanism of ferroptosis in breast cancer has been extensively studied. Research has found that the sensitivity and resistance to ferroptosis are closely related to iron metabolism, antioxidant defense, and tumor microenvironment factors. By targeting key ferroptosis regulators such as GPX4, ACSL4, and SLC7A11, it is possible to effectively overcome chemotherapy resistance and selectively eliminate resistant cancer cell populations (37). Additionally, ferroptosis is also related to the interaction with the tumor immune microenvironment and can recruit immune cells by releasing damage-associated molecular patterns (DAMPs), thereby influencing the immune response of TNBC (36). Secondly, the application potential of ferroptosis in different breast cancer subtypes has also been verified. For example, studies have shown that ATR (a small molecule) induces ferroptosis in triple-negative and luminal-B breast cancer subtypes, demonstrating anti-cancer activity (38). Moreover, atrazaetil (ATR) inhibits the proliferation of MDA-MB-231, MCF-7, BT-474, and SK-BR-3 breast cancer cells, showing significant anti-cancer effects (38). These research results indicate that ferroptosis inducers have broad application prospects in breast cancer treatment. Finally, ferroptosis also shows potential in overcoming drug resistance in breast cancer. It has been pointed out that by inhibiting Xc- system, depleting glutathione (GSH), and inactivating glutathione peroxidase 4 (GPX4), ferroptosis can be effectively induced, thereby reversing tumor resistance (39). Moreover, ferroptosis can also be combined with traditional therapies to enhance the efficacy of existing treatments, especially in breast cancer subtypes resistant to chemotherapy and radiotherapy (40).

Despite the promising guidance offered by our 8-gene risk model and nomogram for the prognosis of BRCA patients, the study had certain limitations. Primarily, the TCGA database presents a geographical bias as it encompasses only patients from the United States. Additionally, due to constraints in clinical specimens and experimental conditions, the roles of the eight genes within BRCA-related signaling pathways have not been thoroughly investigated. Consequently, the specific mechanisms through which these eight genes influence the prognosis of BRCA patients require further exploration and research.

Additionally, the independent validation dataset (GSE162228) used in this study has inherent methodological limitations. Firstly, this dataset contains multiple samples from the same individual, which violates the basic assumption of sample independence in conventional statistical tests. Although we observed consistent significant trends (such as the expression change direction of key gene X) in the analysis, this non-independence between samples may lead to bias in the estimation of statistical significance, potentially underestimating the P-value. Secondly, this dataset integrates data from different gene expression platforms (such as microarrays and RNA-seq). Existing studies have clearly pointed out (as cited in BSR20211428), that technical differences between different platforms introduce significant batch effects, which are unignorable confounding factors in the analysis (14). Although we were unable to perform advanced batch correction in this analysis, we noticed that the core signal still showed commonality in the preliminary exploration of subsets on different platforms. Consistent with the exploratory nature of the GSEA employing an FDR threshold of 0.25, the identified pathways (e.g., Wnt signaling, immune-related complexes, T-cell receptor complex, endothelial cell differentiation) should be regarded as hypothesis-generating and require further experimental validation before being considered confirmatory mechanistic insights. Therefore, the confirmatory conclusions drawn from GSE162228 should be interpreted with caution and regarded as supportive rather than conclusive evidence of the main research findings. Future research is necessary to conduct final validation of the study's findings in a cohort with completely independent samples and a unified platform, or by applying statistical models that can correct for repeated measurements and complex batch effects.

In summary, the diverse functions of *ANO6*, *LIFR*, and *PPARG* in tumors, along with their potential as prognostic biomarkers, underscore their significance as targets in cancer research. Comprehensive investigations into their mechanisms of action across various cancer types, particularly breast cancer, may yield novel insights and strategies for cancer diagnosis and treatment.

## Data Availability

The datasets used and/or analyzed during the current study are available from the corresponding author upon reasonable request.

## Supplementary Materials

Click to view [pdf].
