# Supplementary material for: Identification and validation of prognostic characteristics of survival risk marker genes associated with ferroptosis and iron metabolism in breast cancer
Source: Braz J Med Biol Res. 2026 Oct 30;59:e15313. doi: 10.1590/1414-431X2026e15313 (PMC13722004; doi:10.1590/1414-431X2026e15313)
Supplement: Supplementary file 1 [file 1414-431X-bjmbr-59-e15313-suppl.pdf]

Figure S1. Flowchart illustrating the processes of data collection, processing, analysis, modeling, verification, and additional analytical procedures for survival risk marker genes associated with ferroptosis and iron metabolism in breast cancer (BRCA). DEGs: differentially expressed genes.

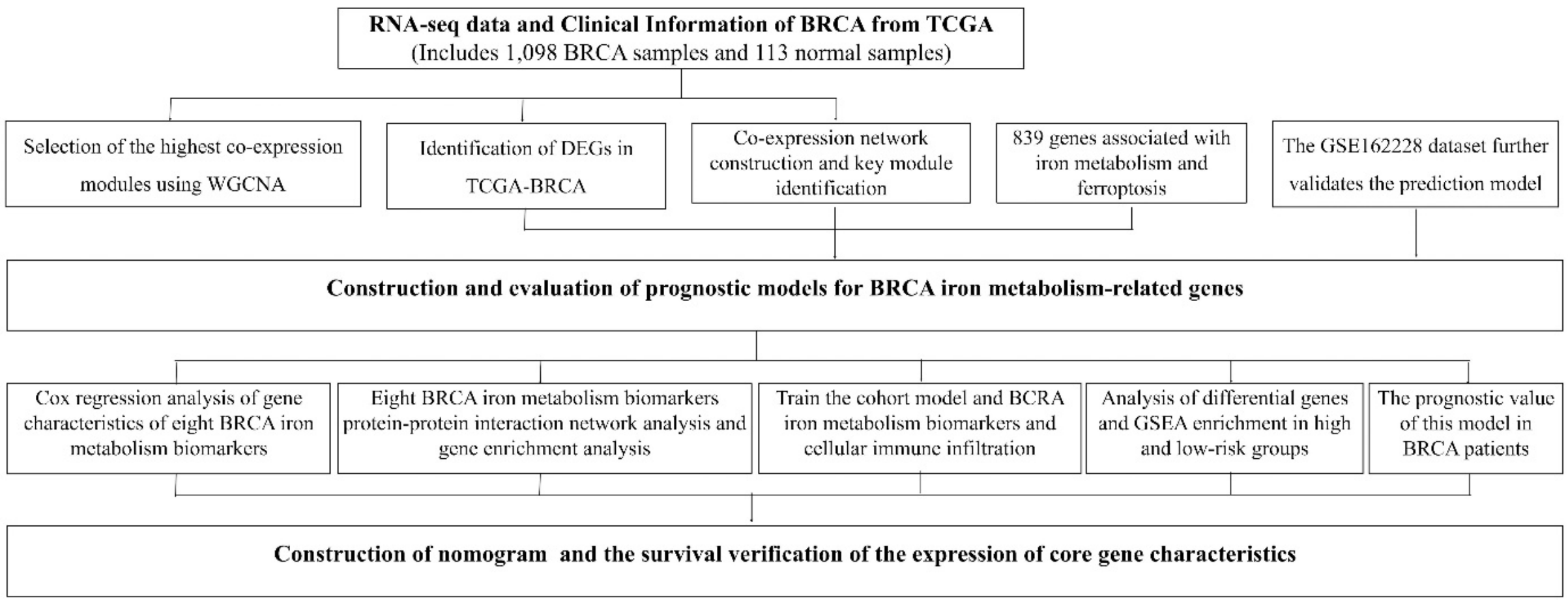

Figure S2. Construction of co-expression network and identification of key modules in breast cancer (BRCA). **A** and **B**, Selection of the "soft" threshold based on a comprehensive analysis of scale independence and average connectivity. **C**, Gene co-expression modules are represented by different colors within the gene tree. **D**, Graphical representation illustrating the relationship between the display module and clinical features, with each cell indicating the correlation and associated P-value. **E**, Collinearity heatmap of the module's characteristic genes, emphasizing high correlation. **F**, Scatter plot depicting the relationship between module membership (MM) in the most relevant modules and gene significance (GS).

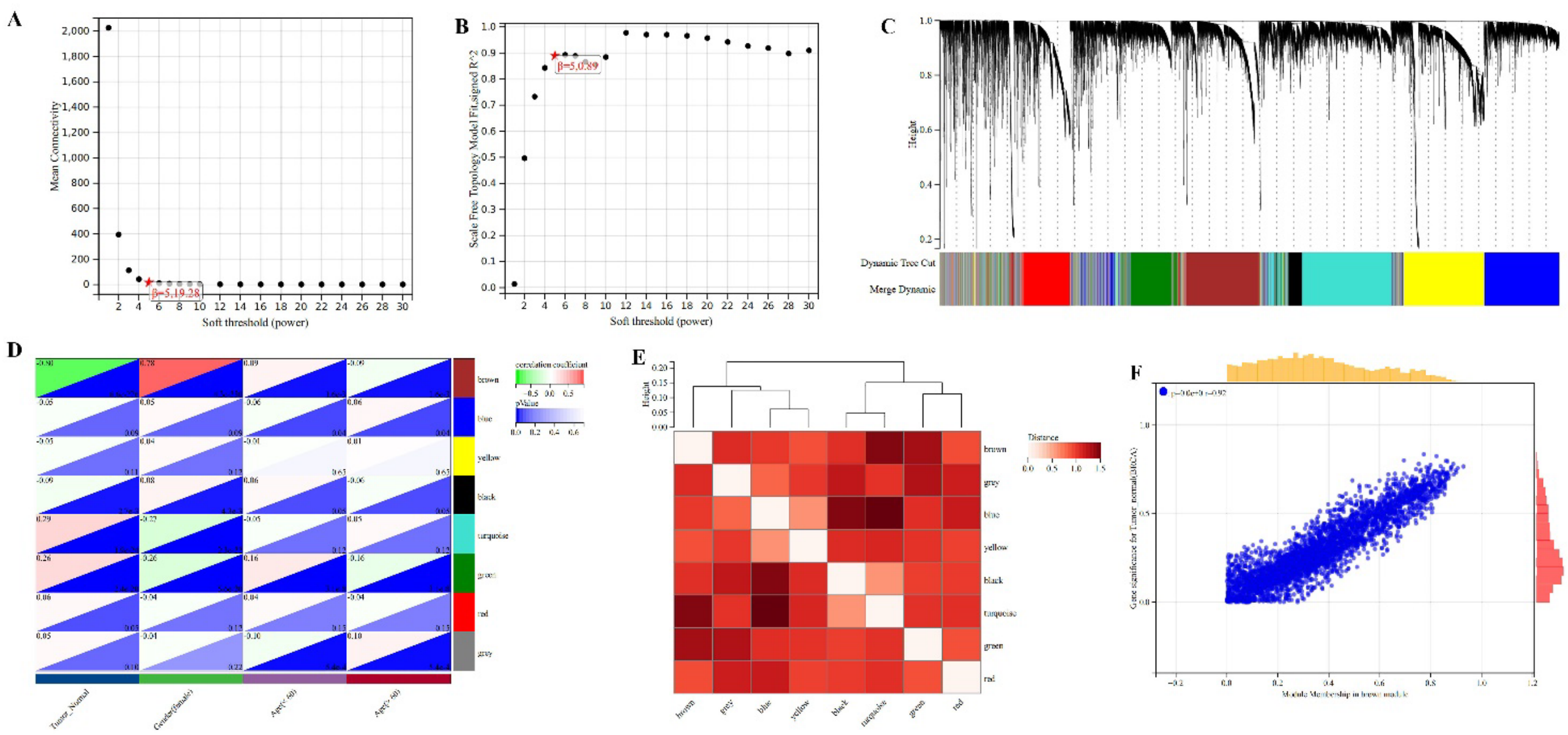

Figure S3. Immune infiltration related to the eight hub genes within the TCGA prediction model. **A**, The proportion of immune cells was compared between the high-risk and low-risk groups in the prognostic model. **B** and **C**, Comparative analysis of immune cells between the high-risk and low-risk groups in the prognostic model: Data are reported as median and interquartile range. ns: P>0.05, *P<0.05, **P<0.01, and ***P<0.001. Mann-Whitney U test followed by Benjamini-Hochberg correction (FDR<0.05). **D**, Correlation analysis between the eight hub genes and tumor immune-infiltrating cells.

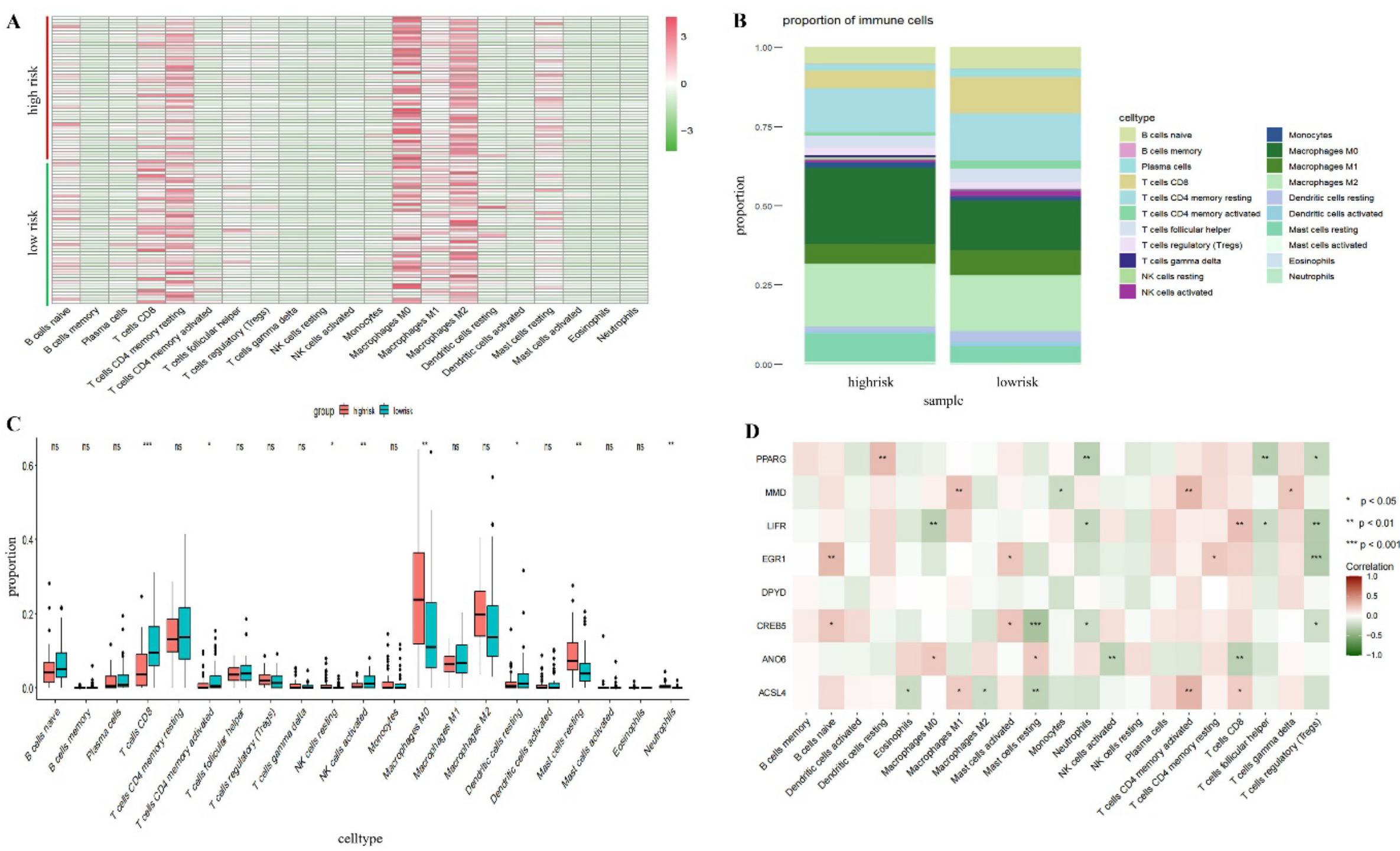

Figure S4. Expression of hub genes in breast cancer (BRCA) based on breast cancer subclasses. **A**–**C**, Expression of *ANO6*, *LIFR*, and *PPARG* in BRCA based on breast cancer subclasses. Data are reported as median and interquartile range. Kruskal-Wallis test.

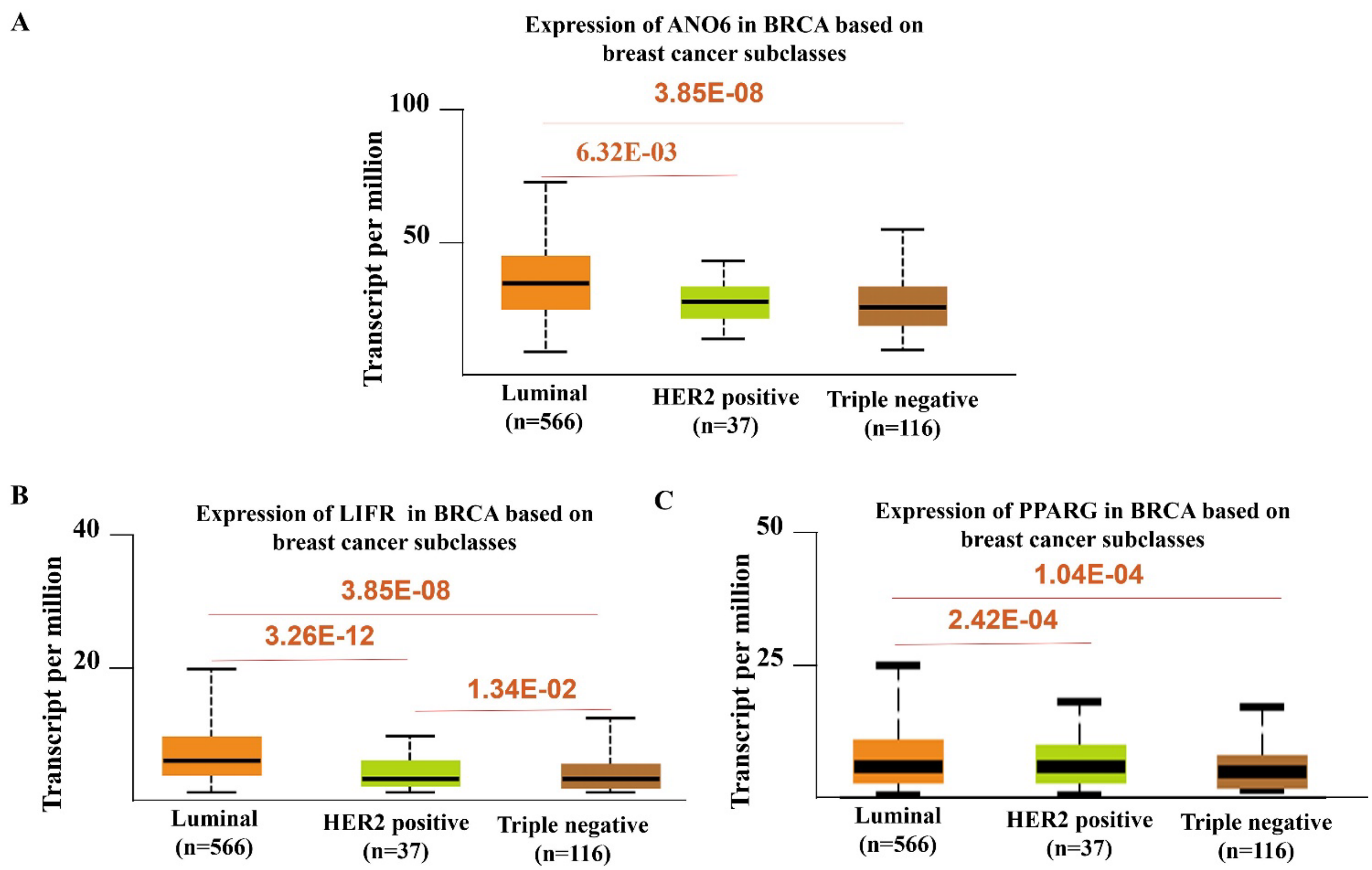

Table S1. Differentially expressed genes (DEGs) were identified from the breast cancer (BRCA) dataset.

Tag	logFC	*t*	P-value	adj.P.Val	B	Change
COL10A1	6.858043006	34.06328352	5.56E-179	7.02E-175	398.5755564	UP
MMP13	6.189085	23.65234822	8.26E-102	1.56E-99	221.4847302	UP
MMP11	5.758067169	33.95590275	3.61E-178	3.42E-174	396.7114299	UP
LINC01614	5.530963324	30.06236551	2.14E-148	5.79E-145	328.3532895	UP
COL11A1	5.315009707	22.60551543	1.07E-94	1.47E-92	205.1639714	UP
PLPP4	5.156541628	27.4941432	1.26E-129	1.01E-126	285.3111432	UP
CST1	5.149592376	11.50700975	4.96E-29	5.12E-28	54.96924896	UP
MMP1	5.033044937	17.48396701	3.60E-61	1.28E-59	128.291661	UP
IBSP	4.888933917	15.65091223	4.01E-50	9.70E-49	103.1074561	UP
DSCAM-AS1	4.687285047	8.077637735	2.14E-15	1.16E-14	24.04362418	UP
SLC24A2	4.642152357	20.99628627	1.61E-83	1.35E-81	179.5353383	UP
COMP	4.553336027	22.12638035	2.20E-91	2.57E-89	197.5647194	UP
NEK2	4.469900963	35.20039986	1.53E-187	5.78E-183	418.2149917	UP
S100P	4.457930365	16.28168101	4.69E-54	1.28E-52	111.9731321	UP
EPYC	4.392572592	15.57557972	8.08E-50	1.94E-48	102.3286456	UP
PITX1	4.243174024	19.3222223	1.06E-72	5.88E-71	154.71203	UP
HOXB13	4.214274929	8.612334409	3.34E-17	1.98E-16	28.03950823	UP
PKMYT1	4.18650702	32.67615808	1.71E-168	1.29E-164	374.5105079	UP
UBE2C	4.144227622	28.60884455	5.56E-138	8.77E-135	304.4899195	UP
GRM4	4.069015443	17.138848	4.14E-59	1.36E-57	123.5589528	UP
TNNT1	4.046804434	13.52666596	6.37E-39	9.84E-38	77.33486749	UP
KIF4A	3.928145723	31.10240643	1.25E-156	4.73E-153	347.2854164	UP
PLAC1	3.821097481	17.5402117	2.60E-61	9.27E-60	128.6566606	UP
GJB2	3.80631025	20.20750484	1.72E-78	1.19E-76	167.9904758	UP
BIRC5	3.798270783	26.76567805	2.18E-124	1.27E-121	273.2954841	UP
MS4A15	3.767855854	9.183086336	2.13E-19	1.41E-18	33.02762221	UP
IQGAP3	3.759767069	29.48695233	1.60E-144	3.38E-141	319.497136	UP
CST2	3.755802796	14.37977726	3.12E-43	5.63E-42	87.28332582	UP
FOXD3-AS1	3.704200348	13.94000751	7.23E-41	1.21E-39	81.84100748	UP
KIF18B	3.694635434	26.95292742	9.71E-126	6.45E-123	276.3940185	UP
LINC01705	3.654228356	12.41523442	3.34E-33	4.07E-32	64.51901793	UP
CST4	3.652112351	6.975430384	5.50E-12	2.46E-11	16.46298428	UP
LINC00922	3.621030836	17.23803829	1.65E-59	5.50E-58	124.5329258	UP
UHRF1	3.615886883	30.18186593	1.02E-149	3.21E-146	331.4257915	UP
MYBL2	3.609158728	21.72235342	1.23E-88	1.31E-86	191.2575651	UP
ASPM	3.605919167	25.74463999	6.14E-117	2.30E-114	256.1971045	UP
TOP2A	3.5936327	25.68198778	1.67E-116	6.10E-114	255.1984733	UP
NUF2	3.589408659	27.81153968	4.46E-132	4.12E-129	290.9395491	UP
H2AC13	3.579603628	14.7595767	2.63E-45	5.20E-44	92.01667214	UP
CDC25C	3.575322219	29.86897461	2.24E-147	5.65E-144	326.0496332	UP
TROAP	3.555675795	27.29757035	2.73E-128	2.15E-125	282.2486881	UP
CCL11	3.554815881	20.63290652	4.96E-81	3.82E-79	173.8324261	UP
HJURP	3.554524192	28.14372488	1.56E-134	1.74E-131	296.5755176	UP
PBK	3.539935885	27.2120164	1.16E-127	8.63E-125	280.8056879	UP
LRRC15	3.536630456	19.03447029	7.03E-71	3.67E-69	150.5343063	UP
KIF14	3.532338965	24.32278634	9.46E-107	2.24E-104	232.8201896	UP
NKAIN1	3.529080524	12.65371809	1.47E-34	1.90E-33	67.3473215	UP
RRM2	3.520229369	26.74697522	2.98E-124	1.71E-121	272.9814652	UP
MELK	3.517723	25.88055768	6.10E-118	2.38E-115	258.4988522	UP
CNTNAP2	3.510211957	11.84162572	1.10E-30	1.22E-29	58.49677993	UP
CD36	–4.749017115	–23.40574642	2.84E-100	4.91E-98	217.9570541	Down
SFRP1	–4.776098859	-17.11639019	5.40E-59	1.77E-57	123.2893942	Down
KCNIP2	–4.779913837	–28.06656819	5.81E-134	5.95E-131	295.2651861	Down
TIMP4	–4.816698044	–21.1937696	5.35E-85	4.82E-83	182.9161252	Down
WIF1	–4.853279855	–14.98715851	3.84E-46	7.89E-45	93.87646358	Down

adj.P.Val: adjusted P-value.

Table S2. Summary of key characteristics for the prognostic core genes.

Gene Symbol	logFC	P-value	adj.P.Val
CAV1	–3.33273512	7.63166079508711e-158	3.61292361615418e-154
PPARGC1B	–1.256440471	5.51109989974687e-31	6.18068956183338e-30
SCARA5	–6.353481209	2.31655353661948e-107	5.69706701898633e-105
LIFR	–2.922343999	2.38881560114335e-101	4.39182588650981e-99
ENPP2	–3.05755818	3.27132683193169e-97	4.99576456071568e-95
PDK4	–3.893926342	1.51784426992642e-96	2.22811302460942e-94
MOCS1	–1.943807458	3.53709995707935e-96	5.11299949139184e-94
CDO1	–3.91667011	3.56347386483117e-92	4.39607314927528e-90
EPAS1	–1.897009504	7.65281210054664e-90	8.49955872973616e-88
ACO1	–1.550829356	1.25810127829617e-89	1.38915655139682e-87
DDR2	–2.46635643	3.42677128143418e-89	3.72937094085508e-87
AKR1C1	–4.256664081	2.19370636963886e-88	2.25766960155795e-86
GABARAPL1	–1.526057686	3.89501797912844e-86	3.71576866306124e-84
MMD	–2.118030183	2.5491396103484e-83	2.12183658159835e-81
FABP4	–6.146693083	5.10867454466179e-80	3.72795435510551e-78
CREB5	–2.380562017	2.54562245583081e-78	1.75931312535913e-76
EGR1	–3.058593615	4.16488652015882e-75	2.54004423797061e-73
ACSL1	–2.331170034	2.80625187882459e-74	1.66584917565397e-72
ANO6	–1.353496593	1.47388900517183e-72	8.13711345377154e-71
SNCA	–2.026438472	2.34826285215684e-72	1.28334428571048e-70
CYP2U1	–1.530845148	9.26025417487632e-72	4.95358201080637e-70
ADIPOQ	–6.389110978	1.52155127412884e-62	5.67740999064842e-61
TYRO3P	–0.421627829	0.00140986990706068	0.00330318608042742
YAP1	–1.234415411	8.29779875217444e-50	1.98523393645675e-48
TLR4	–1.579768631	7.29496301035056e-49	1.68362056118834e-47
DPYD	–1.501962811	6.61450241129486e-44	1.23101252984261e-42
PTGIS	–2.12455372	2.02222304681251e-43	3.68209872365048e-42
ACSL4	–1.381378243	4.75632305724488e-42	8.24422073899475e-41
CYGB	–1.35862165	2.707318101079e-38	4.08503021681932e-37
RBMS1	–1.060736082	2.52736175440116e-37	3.63949702374278e-36
ZEB1	–1.040639197	1.02932997393816e-21	7.57997552069994e-21

adj.P.Val: adjusted P-value.
